# Access to resources buffers against effects of current reproduction on future ability to provide care in a burying beetle

**DOI:** 10.1002/ece3.9266

**Published:** 2022-09-09

**Authors:** Georgia A. Lambert, Per T. Smiseth

**Affiliations:** ^1^ Institute of Ecology and Evolution University of Edinburgh Edinburgh UK

**Keywords:** cost of reproduction, life‐history trade‐offs, *Nicrophorus vespilloides*, parental care, reproductive allocation

## Abstract

Studies investigating the trade‐off between current and future reproduction often find that increased allocation to current reproduction is associated with a reduction in the number or quality of future offspring. In species that provide parental care, this effect on future offspring may be mediated through a reduced future ability to provide care. Here, we test this idea in the burying beetle *Nicrophorus vespilloides*, a species in which parents shift the cost of reproduction toward future offspring and provide elaborate parental care. We manipulated brood size to alter the costs females experienced in association with current reproduction and measured the level of parental care during a subsequent breeding attempt. Given that these beetles breed on carcasses of small vertebrates, it is important to consider confounding effects due to benefits associated with resource access during breeding. We, therefore, manipulated access to carrion and measured the level of parental care during a subsequent breeding attempt. We found that females provided the same level of care regardless of previous brood size and resource access, suggesting that neither affected future ability to provide care. This may reflect that parents feed on carrion during breeding, which may buffer against any costs of previous breeding attempts. Our results show that increased allocation to current reproduction is not necessarily associated with a reduction in future ability to provide care. Nevertheless, this may reflect unique aspects of our study system, and we encourage future work on systems where parents do not have access to a rich resource during breeding.

## Graphical abstract

We investigated whether females of the burying beetle *Nicrophorus vespilloides* that invested more during a current breeding attempt were less able to provide care during a subsequent breeding attempt. Our results showed that females maintained the level of care they provided regardless of previous reproductive allocation. This may reflect that females feed on carrion during breeding, which may buffer against costs of previous breeding attempts.

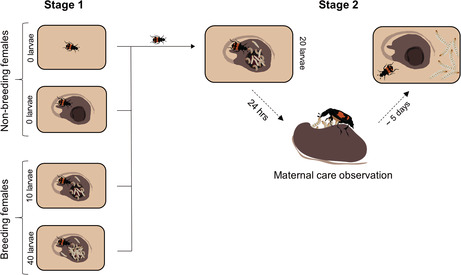

## 1 INTRODUCTION

The trade‐off between current and future reproduction, often referred to as the “cost of reproduction,” is a central hypothesis to life‐history theory (Williams, 1966). Allocation to current reproduction diverts resources from somatic maintenance, which, in turn, may reduce future reproductive potential. The cost of reproduction is often studied experimentally by altering parental allocation to current reproduction and then measuring its effect on future breeding attempts. This is usually achieved by manipulating brood size (Koivula et al., 2003; Lessells, 1986; Parejo & Danchin, 2006), but can also involve manipulating the physiology of mothers (Cox & Calsbeek, 2010; Oksanen et al., 2002) or the reproductive environment (Creighton et al., 2009). Currently, evidence for such a trade‐off is mixed with some studies supporting the hypothesis but others finding either no relationship or a positive relationship between current and future reproduction (Santos & Nakagawa, 2012). Within studies reporting evidence for a cost of reproduction, this cost can manifest in different ways. In some species, the cost to future reproduction is paid through a reduction in the parents' own survival, for example, as a result of increased susceptibility to predation (Veasey et al., 2001) or parasitism (Alt et al., 2015). Meanwhile, in other species, the cost is shifted toward future offspring as indicated by a reduction in the number or quality of offspring produced in subsequent breeding attempts (Martin & Festa‐Bianchet, 2010; Nur, 1988; Parejo & Danchin, 2006). In such species, little attention has been given to how the parents are able to shift the cost of reproduction onto future offspring and this therefore remains poorly understood.

In species where parents care for their offspring, parents may shift the cost of reproduction onto future offspring if increased allocation to reproduction is associated with a reduction in future ability to provide care. Parents that allocate more toward current reproduction may reduce the level of care they provide during subsequent breeding attempts either because they cannot allocate as much toward care as a result of higher investment in current reproduction, or because they strategically allocate less toward care in order to maintain their own condition and facilitate future breeding opportunities. For example, there is observational evidence that bighorn sheep (*Ovis canadensis*) ewes reduce the energy they allocate to their lambs via lactation and instead prioritize their own body mass when resources are limited (Martin & Festa‐Bianchet, 2010). By doing so, ewes increase their own over‐winter survival at the expense of that of their lambs. Such reduction in parental care may provide a general mechanism for the commonly observed reduction in number or quality of future offspring associated with increased allocation to current reproduction in species with parental care. However, little is known about this mechanism and there is now a need for more studies, including those based on an experimental approach, to investigate whether an increase in current reproduction is associated with a reduction in future ability to provide care.

We tested this idea by manipulating reproductive allocation to a current breeding attempt and measuring the effect on the level of care provided in a subsequent breeding attempt by females of the burying beetle *Nicrophorus vespilloides*. Beetles in the genus *Nicrophorus* are well suited to test this idea because there is evidence for a cost of reproduction in *N. vespilloides* (Jenkins et al., 2000; Ward et al., 2009) and the closely related *N. orbicollis* (Billman et al., 2014; Creighton et al., 2009). Studies show that parents shift this cost toward future offspring as parents reduce the size and mass of future broods in response to an increase in allocation to current reproduction induced by manipulating male assistance in parental care (Jenkins et al., 2000), brood size (Ward et al., 2009) or carcass size (Billman et al., 2014; Creighton et al., 2009). *Nicrophorus vespilloides* breeds on a small vertebrate carcass that serves as a food source for caring parents and their offspring. Females (sometimes assisted by a male) provide elaborate parental care for their offspring by preparing the carcass, provisioning pre‐digested carrion to the offspring, maintaining the carcass by spreading antimicrobials onto it, and guarding the offspring and carcass against conspecifics (Eggert et al., 1998; Scott, 1998). Larvae benefit from parental care in terms of increased growth and survival (Andrews et al., 2017; Eggert et al., 1998; Lock et al., 2004). Although previous work provides clear evidence that increased allocation is associated with a reduction in the size and quality of future broods, it remains unknown whether this is due to a reduction in the level of parental care toward future broods.

Our aim was to test whether a reduction in the parent's future ability to provide care due to increased allocation to a current breeding attempt is the mechanism mediating the observed shift in the cost of reproduction toward future broods. We altered female allocation to current reproduction by manipulating whether or not females had the opportunity to breed and, if they did, the size of the brood they cared for. We then measured effects on the level of post‐hatching care provided in a future breeding attempt. If increasing the cost of reproduction in an initial breeding attempt affected a female's ability to provide care in a future breeding attempt, we predict that breeding females that had cared for a large brood would provide the lowest level of care during the subsequent breeding attempt, that non‐breeding females would provide the highest level of care, while breeding females that had cared for a small brood would provide an intermediate level of care. In *N. vespilloides*, parents have access to a nutrient‐rich carcass that they feed on during reproduction, which often leads to an increase in mass over a breeding attempt (Creighton et al., 2009; Pilakouta et al., 2016; Richardson et al., 2019, 2020; Richardson & Smiseth, 2019). Thus, when studying potential effects associated with a cost of reproduction, it is important to consider confounding effects due to potential benefits associated with gaining access to resources during breeding. Accordingly, we investigated whether prior access to a mouse carcass affected future post‐hatching parental care. We, therefore, included two control groups. First, non‐breeding females that had no prior carcass access and did not produce a brood and, therefore, had neither the benefits of carcass access nor the costs of reproducing. Second, non‐breeding females that had access to a carcass prior to their first breeding attempt but did not produce a brood and, therefore, had the benefits of carcass access but suffered none of the costs of caring for larvae. If the benefit associated with carcass access in an initial breeding attempt affected a female's ability to provide care in a future breeding attempt, we predict that breeding and non‐breeding females that had prior access to a carcass would provide more care than non‐breeding females that did not have such access. Finally, we tested for combined effects of the cost of reproduction and the benefit of carcass access on the level of post‐hatching care females provided during a subsequent breeding attempt. If there were such combined effects, we predicted that the pattern observed would be intermediate between those described above for what we predicted if there were effects due to the cost of reproduction or the benefit of carcass access.

## 2 MATERIALS AND METHODS

### 2.1 General methodology

We used beetles from an outbred laboratory population maintained at the University of Edinburgh. All beetles in the stock population were originally collected in Edinburgh, UK and the population was kept at 20°C under a 16:8 h light: dark cycle. We housed non‐breeding adults individually in clear plastic containers (12 cm × 8 cm × 2 cm) lined with moist soil and fed them raw organic beef twice a week.

### 2.2 Experimental design

**FIGURE 1:**
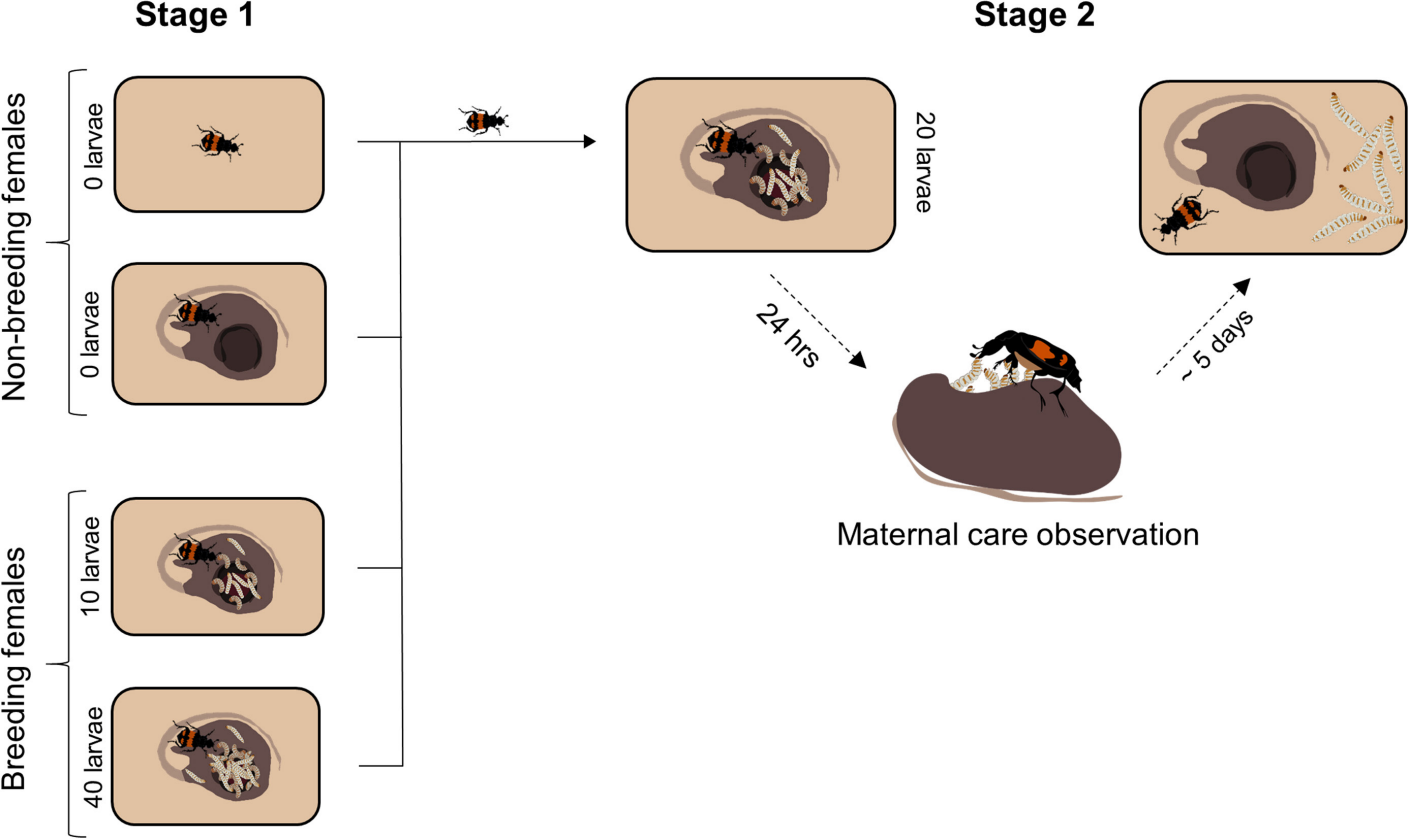
The experimental design used to investigate whether the cost of increased reproductive allocation and benefit of carcass access (manipulated during stage one) affect the level of care females provide during stage two.

The experiment was conducted across two stages. In stage one, we randomly assigned unmated females to one of the following four treatments (Figure 1): breeding females allocated a large brood of 40 larvae (*n* = 32), breeding females allocated a small brood of 10 larvae (*n* = 32), non‐breeding females provided with access to a mouse carcass (*n* = 30) or non‐breeding females not provided with access to a mouse carcass (*n* = 30). We chose these brood sizes to represent a low and high level of reproductive allocation, respectively, since they are approximately half and double the average brood size for *N. vespilloides* (21 larvae; Smiseth & Moore, 2002) and fall well within the natural range for this species (2–45 larvae; Smiseth & Moore, 2002). We included the treatment of non‐breeding females provided with access to a carcass to control for any benefits associated with carcass access. The extent of the benefit associated with carcass access is unclear because *N. vespilloides* feeding habits in the absence of a carcass in the wild are unknown. However, they are suspected to feed on other insects as well as carrion, and carcasses would be of comparatively high nutritional value. Carcass access also stimulates mated and unmated females to lay fertilized or unfertilized eggs, respectively, and to provide pre‐hatching parental care (i.e., burying and maintaining the carcass). It is not possible experimentally to separate the benefit of feeding from the carcass from the costs of egg laying and pre‐hatching care. However, the cost of egg laying and providing pre‐hatching care is known to be minimal (undetectable in Ward et al., 2009). Accordingly, we expected the net effects of carcass access to be beneficial.

In stage two, all females from stage one were provided with a carcass to initiate breeding and all females were allocated the same, standardized brood size (20 larvae; Figure 1). We used a standardized brood size to control for potential confounding effects due to variation in clutch size or brood size, which might affect the amount of care provided by females. This design allowed us to test whether the observed shift in the cost of reproduction toward future broods was mediated by a reduction in the parent's future ability to provide care while controlling for any potential benefit associated with carcass access. We note that this design excludes the possibility to test how treatment during stage one would affect the natural broods produced by the females during stage two. However, prior work on this species has established that increased allocation is associated with a reduction in the size and quality of future broods in *N. vespilloides* (Jenkins et al., 2000; Ward et al., 2009). If the observed shift in the cost of reproduction toward future offspring was mediated by a reduction in the parent's future ability to provide care, this would be detected as a reduction in the amount of care provided during stage two in this descending order: breeding females that had cared for a large brood during stage one, breeding females that had cared for a small brood, non‐breeding females that had not had access to a carcass. If there was a benefit associated with carcass access, and this benefit affected the parent's future ability to provide care, this would be detected as an increase in the amount of care provided during stage two by non‐breeding females that had access to a carcass during stage one compared to non‐breeding females that had not had access to a carcass.

In stage one of the experiment, breeding females were weighed and then paired with an unrelated male from the stock population. We transferred each pair into a clear plastic container (17 cm × 12 cm × 6 cm) lined with 1 cm of moist soil containing a freshly thawed mouse carcass (Livefoods Direct Ltd) of a standardized size (20–25 g; M ± *SE* = 22.57 ± 0.19 g) to initiate mating. After 48 h, when the eggs had been laid but before the larvae had begun hatching, we moved the female and her carcass into a new container lined with fresh moist soil. At this point, we discarded the male since males sometimes assist with parental care, in which case the male might absorb some of the cost of reproduction if present. In addition, male involvement in providing care is highly variable (Smiseth & Moore, 2002) and so allowing the male to assist would generate variation in the extent to which males assisted. Therefore, we removed the male to make it easier to detect any cost of reproduction to the female. Removing the male has also been shown to have no effect on female caring behavior or offspring fitness in *N. vespilloides* (Smiseth et al., 2005). We then allocated breeding females either a small (10 larvae) or a large (40 larvae) foster brood which consisted of newly hatched larvae from at least two different mothers. We only allocated a foster brood to a female after her own eggs had started hatching since females use temporal kin recognition and kill larvae that arrive at the carcass before their own eggs have begun to hatch (Müller & Eggert, 1990). We left the females undisturbed to care for their brood until the larvae dispersed from the carcass approximately 5 days later upon which we recorded average larval mass, the proportion of larvae that survived to dispersal and female mass.

Non‐breeding females were also weighed before being transferred into clear plastic containers (17 cm × 12 cm × 6 cm) lined with 1 cm of moist soil. Non‐breeding females provided with access to a carcass were given a freshly thawed mouse carcass (Livefoods Direct Ltd) of a standardized size (20–25 g; M ± *SE* = 22.53 ± 0.24 g). Being provided with a carcass initiated pre‐hatching parental care (preparing the carcass for larvae) and the laying of unfertilized eggs. Non‐breeding females without access to a carcass were fed raw organic beef twice a week (approximately 0.3 g) to ensure they did not starve. Unlike the presence of a carcass, the presence of beef did not initiate egg laying or pre‐hatching care. We handled non‐breeding females an equal number of times as we handled the breeding females described above. Non‐breeding females that were provided access to a carcass had access to it for the same period as the breeding females. We then weighed the females at the end of stage one before they were transferred into individual clear plastic containers (12 cm × 8 cm × 2 cm) lined with moist soil and left undisturbed for 24 hours before proceeding to stage two.

In stage two, we weighed all females used in stage one before pairing them with an unrelated male from the stock population. We then transferred each pair into a clear plastic container (17 cm × 12 cm × 6 cm) lined with 1 cm of moist soil containing a freshly thawed mouse carcass (Livefoods Direct Ltd) of a standardized size (20–25 g; M ± *SE* = 22.63 ± 0.12 g). We allowed pairs to mate for 48 h after which the male was removed. We removed the male to control for potential confounding effects due to variation in the contribution of males toward caring for offspring as described above for stage one of the experiment. At this point, we recorded the number of eggs each female laid by counting the number of eggs visible through the bottom of the clear container. We chose this method to avoid handling and thereby potentially damaging the eggs. Furthermore, it has been shown that the visible number of eggs is strongly correlated with the actual clutch size when the container is lined with a thin layer of soil as they were here (Monteith et al., 2012). We then allocated each female a foster brood consisting of 20 larvae using the same methods as in stage one. We conducted behavioral observations 24 h (±10 min) after we allocated females a foster brood, which is when parents of this species provide the highest level of care (Smiseth et al., 2003). We did the behavioral observations under red light using instantaneous sampling every minute for 30 min consistent with established protocols (Smiseth & Moore, 2002). We then left the females to care for their brood until the larvae dispersed from the carcass approximately 5 days later. At the time of dispersal, we recorded average larval mass and the proportion of larvae that survived to dispersal. We weighed the females for a second time after the larvae had dispersed to estimate mass change over stage two.

After stage two, we transferred all females into individual clear plastic containers (12 cm × 8 cm × 2 cm) lined with moist soil, maintained them using the same protocol applied to the stock population, and checked them twice a week until death. We did this to record their lifespan as the number of days from eclosion to the day the female was found dead.

### 2.3 Statistical analyses

All statistical analyses were conducted using R version 3.6.1 (R Core Team, 2021) with the packages car (Fox & Weisberg, 2019), MASS (Venables & Ripley, 2022), and glmmTMB (Brooks et al., 2017). We used zero‐inflated binomial models in our analyses on time spent provisioning food to larvae since the data for this behavior showed minor zero inflation. We used binomial models in our analysis on carcass maintenance and larval survival and linear models to analyze female mass change and mean larval mass at dispersal. In all these models, we included observation level as a random effect to account for over‐dispersion (Harrison, 2015). We used a negative binomial model to analyze data on number of eggs laid during stage two and Cox's proportional hazards to analyze data on female lifespan. All models included female treatment during stage one as a fixed effect with four levels (breeding and caring for a large brood, breeding and caring for a small brood, non‐breeding and having access to a carcass, non‐breeding and not having access to a carcass). We ran pairwise comparisons using a Tukey's test with the Bonferroni correction whenever treatment had a significant effect.

## 3 RESULTS

### 3.1 Female mass change and offspring performance in stage one

**FIGURE 2:**
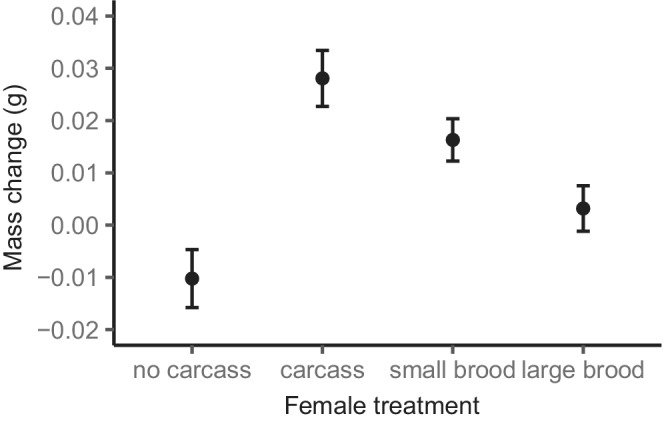
Mean mass change (g) ± *SE* during stage one of non‐breeding females not provided with access to a carcass (no carcass), non‐breeding females provided with access to a carcass (carcass), breeding females allocated a small brood (small brood), and breeding females allocated a large brood (large brood).

Increased reproductive allocation negatively affected female mass change during stage one. Non‐breeding females that had access to a carcass during stage one gained more mass than breeding females that cared for a large brood (estimate ± *SE* = 0.025 ± 0.007 g, *Z* = 3.641, *p* = .002; Figure 2), while breeding females that cared for a small brood gained an intermediate amount of mass midway between breeding females that cared for a large brood (estimate ± *SE* = 0.013 ± 0.007 g, *Z* = 1.950, *p* = .321; Figure 2) and non‐breeding females that had access to a carcass (estimate ± *SE* = 0.012 ± 0.007 g, *Z* = 1.723, *p* = .525; Figure 2). The benefit of carcass access was also evident from effects on female mass change during stage one since non‐breeding females with access to a carcass gained more mass than non‐breeding females that had no access to a carcass (estimate ± *SE* = 0.038 ± 0.007 g, *Z* = 5.516, *p* < .001; Figure 2). There was no difference in mass change between breeding females that cared for a large brood and non‐breeding females that had no access to a carcass (estimate ± *SE* = −0.013 ± 0.007 g, *Z* = −1.963, *p* = .312; Figure 2) and non‐breeding females that had no access to a carcass gained less mass than breeding females that cared for a small brood (estimate ± *SE* = −0.027 ± 0.007 g, *Z* = −3.881, *p* = .001; Figure 2). Thus, our results may suggest that the benefit associated with gaining access to a carcass cancels out or even exceeds the cost of reproduction.

The average mass per larvae at dispersal was higher in small broods than in large broods (*χ* ^2^ = 0.01, df = 1, *p* = .002) but there was no difference in larval survival between small and large broods (*χ* ^2^ = 1.07, df = 1, *p* = .301).

### 3.2 Time spent providing parental care in stage two

**FIGURE 3:**
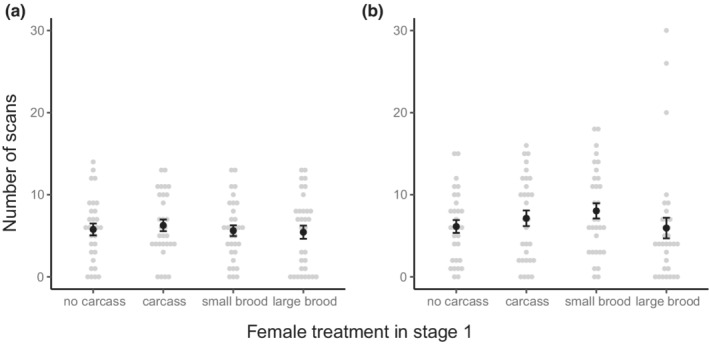
The number of scans during stage two observations in which the female was provisioning food to larvae (a) or maintaining the carcass (b) by females that, during stage one, were non‐breeding and not provided with access to a carcass (no carcass), non‐breeding and provided with access to a carcass (carcass), breeding and allocated a small brood (small brood), and breeding and allocated a large brood (large brood). Gray circles represent individual data, black circles and bars represent means ± *SE*.

There was no evidence that the cost of increased reproductive allocation or the benefit of carcass access during stage one affected the amount of care provided by females during stage two of the experiment (Figure 3). There was no difference in the number of scans during the observations that females from different treatment groups spent provisioning food to larvae (*χ* ^2^ = 1.72, df = 3, *p* = .632; Figure 3) or maintaining the carcass (*χ* ^2^ = 3.35, df = 3, *p* = .340; Figure 3).

### 3.3 Female mass change, egg‐laying and offspring performance in stage two

**FIGURE 4:**
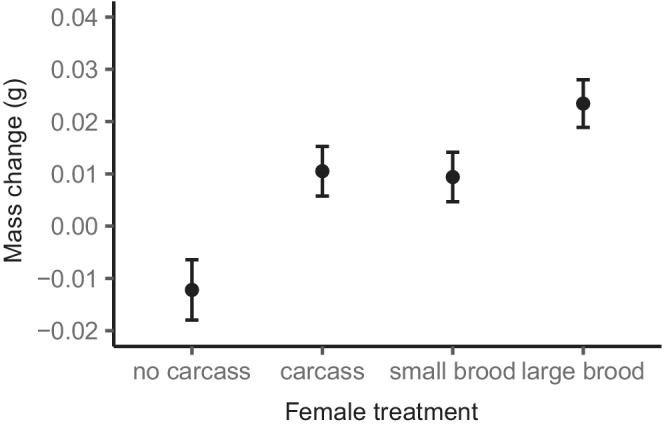
Mean mass change (g) ± *SE* during stage two of females that, during stage one, were non‐breeding and not provided with access to a carcass (no carcass), non‐breeding and provided with access to a carcass (carcass), breeding and allocated a small brood (small brood), and breeding and allocated a large brood (large brood).

Non‐breeding females that had no access to a carcass during stage one gained less mass in stage two than breeding females that had cared for a large brood (estimate ± *SE* = −0.036 ± 0.007 g, *t* = −5.082, *p* < .001; Figure 4), breeding females that had cared for a small brood (estimate ± *SE* = −0.022 ± 0.007 g, *t* = −3.079, *p* = .016; Figure 4) and non‐breeding females that had access to a carcass during stage one (estimate ± *SE* = 0.023 ± 0.007 g, *t* = 3.187, *p* = .011; Figure 4). Thus, females that benefitted from having had access to a carcass during stage one gained more mass during stage two. However, there was no evidence that the cost of caring for larvae in stage one affected female mass gain in a future breeding attempt since there was no difference in mass change in stage two between breeding females that had previously cared for a small brood or a large brood during stage one (estimate ± *SE* = −0.014 ± 0.007 g, *t* = −2.037, *p* = .263; Figure 4). There was also no difference in mass gain between non‐breeding females that previously had access to a carcass and breeding females that previously cared for a small brood (estimate ± *SE* = 0.001 ± 0.007 g, *t* = 0.160, *p* = .999; Figure 4) or a large brood (estimate ± *SE* = −0.013 ± 0.007 g, *t* = −1.844, *p* = .406; Figure 4), further supporting that the cost of caring for larvae in stage one did not affect female mass change during stage two.

There was no evidence that the cost of increased allocation or the benefit of carcass access during stage one affected the number of eggs females laid during stage two since there was no difference in the number of eggs laid by females from the different treatment groups (*χ* ^2^ = 3.946, df = 3, *p* = .267).

There was no evidence that the cost of increased reproductive allocation in stage one affected offspring performance in stage two since there was no difference in larval survival or average larval mass at dispersal in broods reared by breeding females that had previously cared for a large brood or a small brood during stage one (Table 1). There was also no evidence for a benefit associated with access to a carcass during stage one since there was no difference in larval survival or average larval mass at dispersal in broods reared by non‐breeding females that had or did not have access to a carcass during stage one (Table 1). Nevertheless, there were some unexpected effects of our treatment of females during stage one on offspring performance during stage two. Non‐breeding females that had no access to a carcass in stage one produced broods with a higher average larval mass than breeding females that previously cared for a small brood (Table 1). Additionally, broods reared by breeding females that had cared for a large brood during stage one had higher larval survival than broods reared by non‐breeding females that had access to a carcass during stage one (Table 1).

**TABLE 1:** Pairwise comparisons between treatments for offspring performance at dispersal in stage two.

	Proportional larval survival				Average mass per larvae (g)			
	Estimate	*SE*	*Z*	*p*	Estimate	*SE*	*Z*	*p*
Small – large	−0.403	0.289	−1.391	.985	−0.003	0.004	−0.772	1.000
No carcass – large	−0.446	0.293	−1.522	.768	0.010	0.005	2.133	.210
Carcass – large	−0.911	0.289	−3.151	**.010**	−0.002	0.005	−0.367	1.000
No carcass – small	−0.043	0.286	−0.150	1.000	0.013	0.005	2.893	**.027**
Carcass – small	−0.508	0.282	−1.799	.432	0.002	0.005	0.393	1.000
Carcass – no carcass	−0.465	0.286	−1.629	.620	−0.011	0.005	−2.461	.092

*Note*: Statistically significant *p* values (<.05) are shown in bold.

### 3.4 Female lifespan

There was no difference in lifespan between breeding females that cared for a large brood and breeding females that cared for a small brood (Table 2) or non‐breeding females that had access to a carcass during stage one (Table 2). There was also no difference in lifespan between breeding females that cared for a small brood during stage one and non‐breeding females that had access to a carcass during stage one (Table 2). As such, there was no evidence that the cost of caring for a larger brood during stage one had an impact on female lifespan. Furthermore, there was no difference in lifespan between non‐breeding females that did not have access to a carcass during stage one and those that did (Table 2). This finding suggests that the benefit of having access to a carcass during stage one had no effect on female lifespan. However, non‐breeding females that had no access to a carcass during stage one had shorter lifespans than breeding females that cared for a small brood during stage one (Table 2) suggesting that the benefit of having had access to a carcass exceeded the cost of rearing a small brood. There was no difference in lifespan between non‐breeding females that had no access to a carcass and breeding females that cared for a large brood (Table 2) suggesting the cost of rearing a large brood was canceled out by the benefit of having access to a carcass.

**TABLE 2:** Pairwise comparisons between treatments for female lifespan.

	HR	95% CI		*p*
		Lower	Upper	
Large – small	1.496	0.747	2.995	.755
Large – carcass	1.257	0.632	2.504	.999
Small – carcass	1.881	0.948	3.736	.090
No carcass – carcass	1.126	0.565	2.248	.999
No carcass – small	2.119	1.048	4.289	**.029**
No carcass – large	1.418	0.715	2.809	.999

*Note*: Statistically significant *p* values (<.05) are shown in bold.

## 4 DISCUSSION

We found that breeding females that had cared for a large brood provided a similar amount of care during a subsequent breeding attempt as breeding females that had cared for a small brood and non‐breeding females. Thus, our study provided no support for the suggestion that females shift the cost of reproduction as a whole, or the cost of caring for larvae specifically, onto future offspring by providing less care during future breeding attempts. Furthermore, we found that breeding and non‐breeding females that had prior access to a carcass provided a similar amount of care as non‐breeding females that did not have such access. Therefore, there was no evidence that the benefit of carcass access affected the level of post‐hatching care females provided in a subsequent breeding attempt. Our results derive from an experimental design in which we manipulated the cost of reproduction using well‐established methodology (brood size manipulation). We are confident that our treatment had the intended effect since we found that breeding females that had cared for a large brood gained less mass than non‐breeding females that had access to a carcass during stage one, while breeding females that had cared for a small brood gained an intermediate amount of mass. Our design also accounted for potential confounding effects due to the benefit associated with access to carrion by manipulating carcass access during stage one. This benefit was evident since non‐breeding females with access to a carcass gained more mass than non‐breeding females without access to a carcass. Finally, we measured effects on parental food provisioning to larvae and maintenance of the carcass, which are the most predominant parental care behaviors in this species and that are known to impact larval survival and growth (Andrews et al., 2017; Smiseth & Moore, 2002). In sum, we have confidence in our finding that *N. vespilloides* females provided similar levels of post‐hatching parental care regardless of any previous costs associated with reproduction and benefits associated with carcass access.

Our main finding was that breeding females that had cared for a large brood provided a similar amount of care during a subsequent breeding attempt as breeding females that had cared for a small brood and non‐breeding females. Thus, there was no support for the suggestion that a reduction in future ability to provide care is the mechanism allowing parents to shift the cost of reproduction onto future offspring as reported in prior work on beetles in the genus *Nicrophorus* (Billman et al., 2014; Creighton et al., 2009; Jenkins et al., 2000; Ward et al., 2009). There are several potential explanations for the lack of a difference in the amount of care provided by breeding females during stage two of the experiment. First, this could be due to a lack of response to brood size manipulation during stage one. This may be the case if females allocated a large brood during stage one do not to provide more care to maintain their own condition for future breeding opportunities, or if females always work at capacity to provide the highest level of care, in which case they would have little room to escalate the level of care if allocated a large brood. There may appear to be some evidence for this suggestion since average larval mass during stage one was higher in small broods than in large broods. However, we argue that this explanation is unlikely given there is good evidence from prior studies on *N. vespilloides* showing that females provide more care when caring for an enlarged brood (Ratz & Smiseth, 2018; Richardson et al., 2019; Smiseth et al., 2007; Wang et al., 2021). This suggests that breeding females allocated a large brood during stage one would have spent more time providing care than breeding females allocated a small brood. Instead, the finding that average larval mass was higher in small broods than in large broods may simply reflect that there is a trade‐off between number and size of offspring (Richardson & Smiseth, 2019; Smiseth et al., 2014).

Second, the lack of difference in the level of post‐hatching care provided during stage two may reflect the nature of the resources used for breeding by *N. vespilloides*. This species breeds on carcasses of small vertebrates that are used as a food source for both parents and offspring (Scott, 1998). This means that females may gain a benefit associated with feeding from the carcass acquired for breeding. We anticipated such a benefit during both stages of our experiment. In stage one, this benefit was available to breeding females and non‐breeding females provided with a carcass, but not to non‐breeding females not provided with a carcass. Meanwhile, in stage two of our experiment, this benefit was available to all females since we provided all females with a carcass to initiate breeding. Thus, we anticipated seeing effects if the amount of care provided during stage two was affected by carcass access during both stages. Our findings suggest that this was not the case, possibly reflecting that carcass access during stage two had a much greater impact on the females' ability to provide post‐hatching care than either the cost of reproduction or the benefit of carcass access during stage one. In other words, our results suggest that the carcass access associated with reproduction may buffer against any detrimental effects of costs or benefits due to increased allocation to reproduction or carcass access during previous breeding attempts on future post‐hatching parental care.

Our study focused on the amount of care provided by females to larvae, which occurs after the female has had several days to feed and potentially recover from any reduction in condition due to previous allocation. In light of this, it may be important to consider whether the trait in question manifests before or after parents have had access to a carcass when investigating the potential costs of reproduction in *N. vespilloides*. For example while we found no effect of current allocation on the level of future post‐hatching care females provide, there is evidence that increased allocation to current reproduction reduces a female's future ability to compete for a carcass (Richardson et al., 2020). This differential effect on the ability to compete for a carcass and the ability to provide care for larvae may reflect that competition over carcass possession happens before either party has had a chance to feed on the carcass (Safryn & Scott, 2000), while care for larvae happens after females have fed from the carcass. This idea could be investigated by testing the effect of previous reproductive allocation on a trait that could be measured both before and after parents have fed on a carcass during a subsequent breeding attempt such as the ability to defend the carcass from an intruder. Similar results may be expected in other capital breeders that breed on resources obtained prior to reproduction and where parents use these as a food source. In contrast, different results might be expected for income breeders, such as many birds, where parents provision their offspring continuously throughout development with food obtained from the surrounding environment. In the latter species, increased allocation to current reproduction is likely to negatively affect the parents' future ability to provide parental care since parents face a trade‐off between provisioning food to their offspring or consuming food for self‐maintenance. We encourage future studies investigating whether shifting the cost of reproduction toward future offspring is mediated through a reduced future ability to provide care in species with a range of breeding strategies.

Given our finding that females with different levels of previous reproductive allocation provided the same level of care toward their offspring, we must consider alternative mechanisms for the finding that parents shift the cost of reproduction toward future offspring (Billman et al., 2014; Creighton et al., 2009; Jenkins et al., 2000; Ward et al., 2009). One explanation is that greater allocation to current reproduction may affect other aspects of future parental care. In *N. vespilloides*, allocation to current reproduction may affect future egg laying behavior since, like competitive ability, egg laying occurs soon after the discovery of the carcass and when females may have had limited opportunities to recover from any reduction in condition due to previous allocation. Although we found no effect of previous reproductive allocation on the number of eggs females laid during stage two, there may be effects on the pattern of laying or the size of the eggs females produce in a subsequent breeding attempt. For example, females that allocate more to current reproduction may produce smaller eggs or delay the onset of egg laying in future breeding attempts (Mäenpää & Smiseth, 2017). In other species, alternative mechanisms include other behavioral traits, such as a reduction in ability to compete for resources necessary for reproduction (Fokkema et al., 2016) or physiological traits such as a reduction in ornamentation quality limiting breeding opportunities (Siefferman & Hill, 2005). We encourage future work on a variety of taxa investigating alternative mechanisms that could cause increased allocation to current reproduction to result in the often‐observed reduction in future fecundity.

There were some unexpected results from our experiment. First, females that were previously provided access to a carcass (regardless of whether they were breeding or not) gained more mass when provided with a second carcass than females that were not previously provided with a carcass. This finding does not fit our predictions for the effects of the cost of increased allocation or the benefit of carcass access during stage one. Instead, this result suggests that females that previously had access to a carcass responded by shifting toward greater allocation on future reproduction since mass gain during breeding is a proxy for allocation to future reproduction in *N. vespilloides* (Billman et al., 2014; Creighton et al., 2009). This shift may reflect that females responded to the presence of carcasses as an environmental cue about future breeding opportunities. Carcasses are normally a rare resource (Scott, 1998), and coming across two in quick succession may provide females with a cue that they find themselves in a resource‐rich environment. In support of this suggestion, a previous study on *N. vespilloides* found that the quality of the carcass used for a breeding attempt influences reproductive investment in a subsequent breeding attempt, potentially by providing information about the resources available (Billman et al., 2014).

Second, breeding females that were allocated a small brood in stage one produced broods with a lower average larval mass in stage two than non‐breeding females that did not have access to a carcass in stage one. This finding appears to contrast with previous studies, which found that increased allocation to current reproduction is associated with a reduction in the size and mass of future broods (Billman et al., 2014; Creighton et al., 2009; Jenkins et al., 2000; Ward et al., 2009). However, we note that we provided females with foster broods of a standardized size during stage two. This was an important aspect of our design as it allowed us to control for any potential confounding effects on female behavior due to variation in brood size during stage two. In contrast, prior studies manipulated aspects of reproductive allocation during a current breeding attempt and then allowed individuals to raise their natural brood during a subsequent breeding attempt or throughout multiple breeding attempts for the rest of their lifetime. Our results are therefore not comparable with those of prior studies. We also found that breeding females that were allocated a small brood in stage one had a longer lifespan than non‐breeding females that were not provided access to a carcass in stage one. This may appear to contrast with the results of Creighton et al. (2009), which showed non‐reproducing females to live longer than reproducing females. However, Creighton et al. (2009) compared the lifespan of reproducing females and females that never reproduced, whereas we compared females that did not reproduce initially but were allowed to reproduce during stage two with females that reproduced across both stages.

Why did breeding females allocated a small brood in stage one produce broods with a lower average larval mass in stage two and have a longer lifespan than non‐breeding females without access to a carcass in stage one? We suggest that these results reflect the combined effects of breeding females shifting toward greater allocation in future reproduction, breeding females caring for a small brood in stage one suffering a lower cost of caring for larvae than breeding females caring for a large brood, and non‐breeding females with access to a carcass responding to this treatment as a failed breeding attempt. As discussed above, breeding females may have shifted toward greater allocation in future reproduction if the presence of carcasses acts as an environmental cue about abundant future breeding opportunities. However, this alone cannot explain our finding since we would also expect breeding females that cared for a large brood and non‐breeding females with access to a carcass to respond similarly, which was not the case. We therefore suggest that breeding females caring for a small brood suffered a lower cost of caring for larvae than breeding females caring for a large brood. Finally, we suggest that non‐breeding females with access to a carcass responded differently from breeding females given that they produced no larvae during stage one, and therefore may have perceived this as a failed breeding attempt.

In conclusion, our study shows that females maintain the level of parental care they provide regardless of any costs associated with previous reproductive allocation or any benefits associated with prior resource access. This is likely a result of the breeding strategy of *N. vespilloides* since they have access to a fixed food source during breeding which may facilitate their recovery from any costs associated with previous reproductive allocation. Our findings highlight the need for more work exploring the potential mechanisms that allow parents to shift the cost of current reproduction toward future offspring in species with a variety of life‐history strategies.

## AUTHOR CONTRIBUTIONS

**Per T. Smiseth:** Conceptualization (supporting); formal analysis (supporting); methodology (supporting); supervision (lead); writing – review and editing (equal). **Georgia A. Lambert:** Conceptualization (lead); data curation (lead); formal analysis (lead); methodology (lead); writing – original draft (lead); writing – review and editing (equal).

## CONFLICT OF INTEREST

None.

### OPEN RESEARCH BADGES

This article has earned an Open Data badge for making publicly available the digitally‐shareable data necessary to reproduce the reported results. The data is available at https://doi.org/10.5061/dryad.gtht76hqc.

## Data Availability

Data are available from the Dryad Digital Repository: https://doi.org/10.5061/dryad.gtht76hqc.
